# The Oncoprotein SKI Acts as A Suppressor of NK Cell-Mediated Immunosurveillance in PDAC

**DOI:** 10.3390/cancers12102857

**Published:** 2020-10-03

**Authors:** Viviane Ponath, Miriam Frech, Mathis Bittermann, Reem Al Khayer, Andreas Neubauer, Cornelia Brendel, Elke Pogge von Strandmann

**Affiliations:** 1 Institute for Tumor Immunology, Clinic for Hematology, Oncology and Immunology, Philipps University of Marburg, Hans-Meerwein-Strasse 3, 35043 Marburg, Germany; viviane.ponath@staff.uni-marburg.de (V.P.); ms.bittermann@web.de (M.B.); reem.alkhayer@imt.uni-marburg.de (R.A.K.); 2 Clinic for Hematology, Oncology, Immunology and Center for Tumor Biology and Immunology, Philipps University of Marburg, Baldingerstrasse, 35037 Marburg, Germany; frechm@staff.uni-marburg.de (M.F.); neubauer@staff.uni-marburg.de (A.N.); brendelc@staff.uni-marburg.de (C.B.)

**Keywords:** pancreatic tumor, NK cell immunosurveillance, NKG2D, histon (de)acetylation

## Abstract

**Simple Summary:**

Pancreatic ductal adeno carcinoma is one of the most lethal solid tumors and the survival rate has not improved significantly over the past decades. The disease is characterized by an immune-suppressive tumor microenvironment, which promotes the limited response to novel immunotherapies. The aim of our study was to contribute to a better understanding of the diminished Natural Killer (NK) cell-activity in pancreatic cancer. We showed that oncoprotein SKI, which is involved in CBP/p300-mediated acetylation, diminished the expression of activating ligands for the cytotoxicity receptor NKG2D on tumor cells, thereby counteracting NK cell-dependent cytotoxicity. Treatment of tumor cells with histone deacetylase inhibitors (HDACi) induced the expression of these ligands and improved NK cell-dependent killing. Thus, we unraveled a so far unknown role of SKI in NK cell-mediated immunosurveillance. Our results suggest that the combination of HDACi with NK cell-based immunotherapies may be beneficial for pancreatic cancer patients.

**Abstract:**

Drugs targeting epigenetic mechanisms such as histone deacetylase inhibitors (HDACi) suppress tumor growth. HDACi also induce the expression of ligands for the cytotoxicity receptor NKG2D rendering tumors more susceptible to natural killer (NK) cell-dependent killing. The major acetylases responsible for the expression of NKG2D ligands (NKG2D-L) are CBP and p300. The role of the oncogene and transcriptional repressor SKI, an essential part of an HDAC-recruiting co-repressor complex, which competes with CBP/p300 for binding to SMAD3 in TGFβ signaling, is unknown. Here we show that the siRNA-mediated downregulation of *SKI* in the pancreatic cancer cell lines Panc-1 and Patu8988t leads to an increased target cell killing by primary NK cells. However, the higher cytotoxicity of NK cells did not correlate with the induction of NKG2D-L. Of note, the expression of NKG2D-L and consequently NK cell-dependent killing could be induced upon LBH589 (LBH, panobinostat) or valproic acid (VPA) treatment irrespective of the SKI expression level but was significantly higher in pancreatic cancer cells upon genetic ablation of *SKI*. These data suggest that SKI represses the inducible expression of NKG2D-L. The combination of HDACi with NK cell-based immunotherapy is an attractive treatment option for pancreatic tumors, specifically for patients with high SKI protein levels.

## 1. Introduction

Pancreatic ductal adenocarcinoma (PDAC) represents 85% of all pancreatic cancers and exhibits the poorest prognosis of all solid tumors. Incidence rates in the industrialized world are steadily increasing and the 5-year survival rate is below 10% with a median survival of 6 months [1,2,3]. Reasons for the poor prognosis include delayed diagnosis associated with non-resectable locally advanced or metastatic disease and chemo-resistance. One component which critically contributes to drug resistance is the peritumoral desmoplasia, which impairs intra-tumoral drug delivery [4]. PDAC is moreover characterized by an immune-suppressive tumor microenvironment (TME) producing TGFβ and other factors to counteract immunosurveillance, while promoting tumor growth and metastasis for example, through the recruitment of tumor supporting macrophages [5]. Thus, the response of patients with PDAC to immunotherapy is overall limited [6]. However increasing pieces of evidence suggest that the restoration of NK cell activity in the tumor microenvironment (TME) may interfere with PDAC progression [7,8] exerting direct cytotoxicity and orchestrating the anti-tumor immune response. 

One of the major NK cell receptors involved in the recognition and killing of transformed cells is the cytotoxic receptor NKG2D [9]. NKG2D is expressed on NK cells, CD8+ T cells, some γδ T cells and possibly also some CD4+ T cells [10] and known as a sensor for damaged or dangerous cells. In humans, NKG2D is engaged by several ligands, namely MHC class I polypeptide-related sequence A and B (MICA and MICB) [9] and the UL16-binding proteins 1-6 (ULBP1-6) [11,12,13,14]. Not surprisingly, tumor cells develop mechanisms to escape the innate immune surveillance and these strategies include the release of soluble NKG2D-L to render target cells invisible for an NKG2D-dependent NK cell-attack. Soluble ligands for NKG2D do not only passively block receptor activation but moreover cause a downregulation of receptor surface expression [15].

While NKG2D-L are not expressed on healthy cells, they are upregulated on tumor cells. Their expression is regulated by transcriptional, translational and posttranslational mechanisms [16], although the transcription factors involved are not yet entirely defined. A better understanding of the factors that direct NKG2D-L on the surface of target cells will allow the development of novel therapeutic strategies aiming at an increased NKG2D-L expression on tumor cells and thus a potential killing by NK cells. 

Previously we showed that the major acetyltransferases CBP and p300 have a robust, mandatory and general impact on the up-regulation of mouse and human NKG2D-L in response to cellular stress signals or upon HDAC inhibition [17]. These acetyltransferases interact with the oncogene and transcriptional repressor Sloan-Kettering Institute (SKI), for example, by binding to SMAD3 to modulate TGFβ signaling [18].

Thus, SKI inhibits SMAD association with the p300/CBP coactivators and facilitates the recruitment of histone deacetylases to repress gene transcription [19]. SKI is known to promote pancreatic cancer cell proliferation and SKI overexpression is significantly associated with a decreased patients’ survival time [20,21]. Given that HDAC inhibitors (HDACi) induce surface expression of NKG2D-ligands on tumor cells in a CBP/p300-dependent manner [17] we hypothesize that SKI suppresses NKG2D-ligands on pancreatic cancer cells supporting immune evasion. 

## 2. Results and Discussion

**Figure 1:**
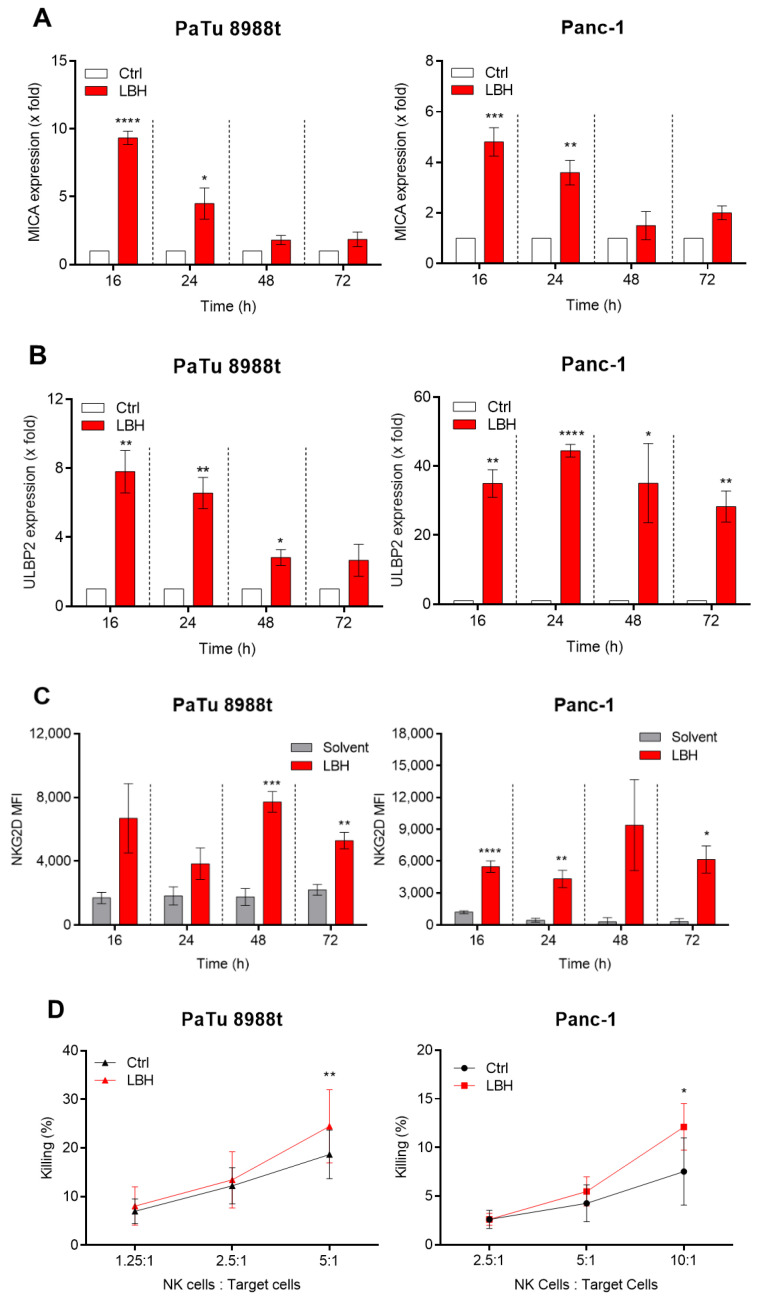
NKG2D ligand expression in PaTu 8988t and Panc-1 cells after treatment with HDACi LBH589. The mRNA of NKG2D ligands (**A**) MICA and (**B**) ULBP2 was measured in PaTu 8988t (left panel) and Panc-1 (right panel) 16, 24, 48 and 72 h after treatment with 100 nM LBH58th 25 nM LBH for up to 72 h and NKG2D-L and the mean fluorescence intensity (MFI) was measured by flow cytometry using recombinant human NKG2D Fc chimera protein. (**D**) Tumor cells were treated with 100 nM LBH589 for 16 h before they were co-cultured with primary NK cells for 3 h and NK cell-mediated killing of target cells was measured by flow cytometry. Data are the mean of three (**A**,**B**,**D**) or four to five independent experiments (**C**) ± SEM. Statistical significance between treated and untreated samples of the individual time points was calculated using Student’s *t*-test (* *p* < 0.05, ** *p* < 0.01, *** *p* < 0.001, **** *p* < 0.0001).

The effect of HDACi is reported to induce NKG2D-L on the surface of tumor cells, which promotes tumor cell recognition and killing by NK cells supporting an anti-tumor immune response. The cellular responses and the affected ligands vary depending on the HDACi applied and the tumor type. Data for pancreatic cancer cells are rare, however the upregulation of MICA/B upon treatment of tumor cell lines with VPA (an HDACi originally developed for the treatment of epilepsy) through the induction of the PI3K/AKT signaling pathway was described [22]. Treatment of the pancreatic cancer cell lines PaTu8988t and Panc-1 with the pan-HDACi panobinostat (LBH589) and subsequent qRT-PCR analysis established that LBH induced the transcription of the ligands *MICA* and *ULBP2* (Figure 1A,B). Induced transcription was most pronounced after 16 h treatment and declined after 48 h and 72 h. The up-regulation of the mRNAs was in line with the enhanced surface expression of ligands for NKG2D measured by flow cytometry (Figure 1C). Here, a similar efficacy for LBH and VPA both used in sublethal concentrations was observed, although the induction efficacy of LBH for MICA and ULBP2 was more robust under the conditions used (Figure S1). 

To test, whether the HDACi-mediated NKG2D ligand induction actually is of biological significance, flow cytometry (FC)-based NK cell killing assays using primary NK cells were performed. Indeed, NK cells were significantly more potent in lysing LBH-treated pancreatic cell lines compared to untreated controls (Figure 1D), manifesting functional relevance of LBH-induced NKG2D ligand upregulation. 

We and others showed that enhanced expression of NKG2D-L results in increased in vitro NK cell killing activity (Figure 1D, [23,24,25]. Xenograft mouse models moreover demonstrated that the pharmacological increase in NKG2D ligand expression also results in enhanced NK cell-mediated tumor surveillance [23,24,25]. The same holds true for syngeneic mouse models where NKG2D ligand induction enables NK cells to reject MHC class I-bearing tumors [26]. Moreover, bifunctional recombinant proteins which are applied to retarget NK cells to tumor cells triggering NKG2D activation reveal potent anti-tumor activity in vivo and in vitro [27]. These observations highlight the promising role of the NKG2D-NKG2D-L axis as a therapeutic target to overcome NK cell anergy in tumor patients. 

**Figure 2:**
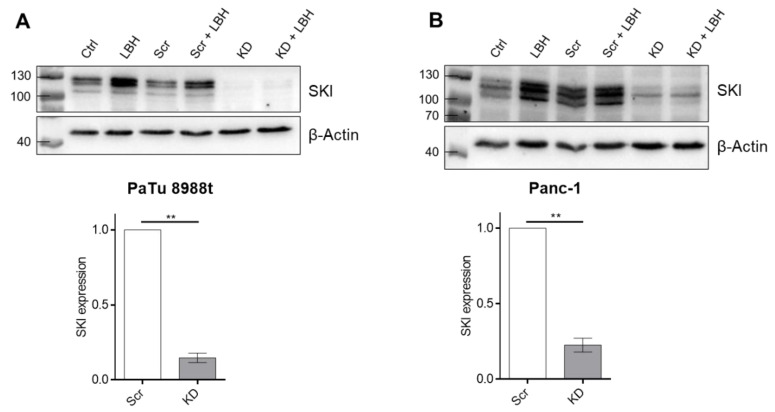
siRNA-mediated knockdown (KD) of SKI. (**A**) PaTu8988t and (**B**) Panc-1 cells were treated with siRNA for SKI knockdown for 24 h followed by 16 h of 100 nM LBH589 treatment before the knockdown was quantified by Western Blot analysis. Ctrl: non treated, scr: scrambled siRNA. The lower panel depicts the quantification of qRT-PCR data for scrambled siRNA-treated sample and siRNA treated samples. Data are the mean of three independent experiments ± SEM. Statistical significance was calculated using Student’s *t*-test (** *p* < 0.01). KD = knockdown, Scr = scrambled.

Having demonstrated that HDACi can induce NKG2D-L and trigger NK cell cytotoxicity we analyzed whether the oncoprotein SKI was involved in NKG2D-ligand regulation. siRNA targeting *SKI* was used to effectively down-regulate SKI in PaTu8988t (upper panel Figure 2A) and Panc-1 (upper panel Figure 2B) pancreatic adenocarcinoma cells and the expression level was determined using Western blotting (Figure 2, see Figure S2 for quantification) and qRT-PCR (lower panel, Figure 2A,B). 

**Figure 3:**
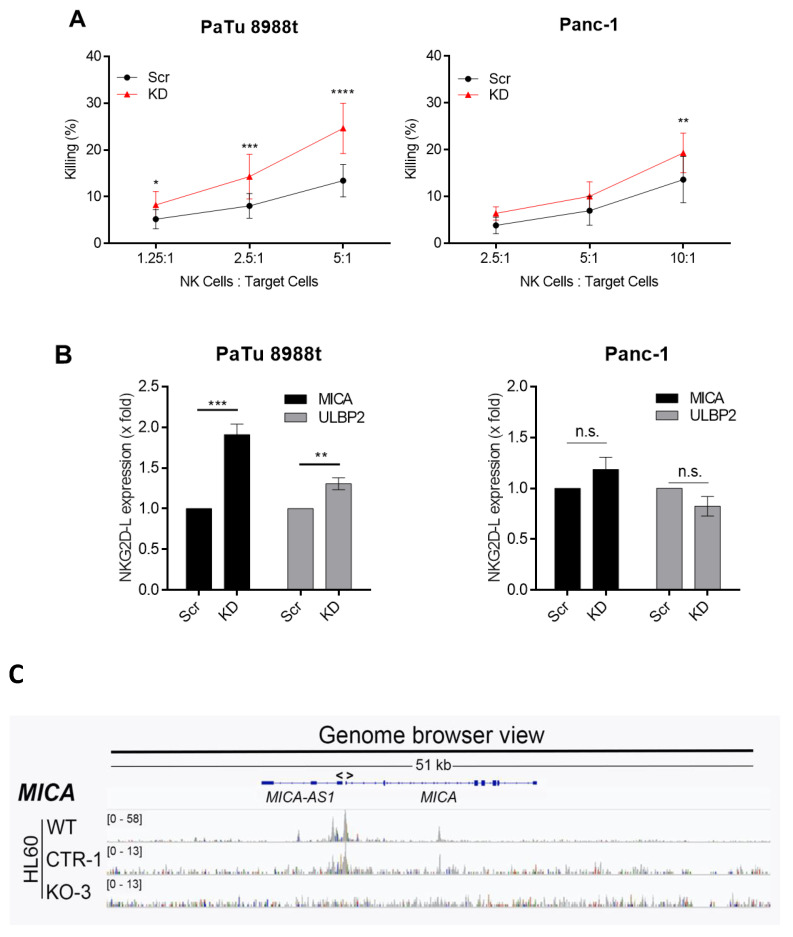
Effect of SKI knockdown on NK cell-mediated killing of target cells and expression of NKG2D-L. (**A**) SKI knockdown was performed in PaTu89888t (left panel) and Panc-1 (right panel) cells for 24 h. Then, cells were co-cultured with primary NK cells for 3 h before target cell killing was measured by flow cytometry. (**B**) The mRNA expression of NKG2D-L *MICA* and *ULBP2* was measured in SKI knockdown cells. Data are the mean of four (**3B**) to five (**3A**) independent experiments ± SEM. Statistical significance was calculated using 2-way ANOVA and Sidak’s multiple comparisons test (**3A**) and Student’s *t*-test (**3B**) (* *p* < 0.05, ** *p* < 0.01, *** *p* < 0.001, **** *p* < 0.0001, n.s.: not significant). (**C**) Genome browser view of the *MICA* genomic region of the SKI ChIPseq analyses of the cell line HL60 (WT), HL60 control cells (CTR-1, CRISPR-Cas9) and HL60-SKI deficient cells (KO-3, CRISPR-Cas9).

Of note, a cytotoxicity assay using primary purified NK cells from healthy donors as effector cells showed that the knockdown of SKI was associated with a significantly better killing efficacy (Figure 3A). The analysis of NKG2D-L expression revealed that *MICA* and *ULBP2* were significantly higher expressed in PaTu8988t cells with diminished SKI expression (Figure 3B), whereas the ligand expression in Panc-1 cells was not affected. We conclude that the increased expression of *MICA* and *ULBP2* in PaTu8988t cells upon SKI knockdown may contribute to a better NK cell-mediated killing but SKI seems to suppress susceptibility against NK cell-mediated killing also by additional, not yet defined mechanisms. In this regard, we found in acute myeloid leukemia (AML) cells that SKI acts rather as a transcriptional repressor and inhibits target genes associated with inflammatory response, immune cell differentiation as well as TNFα signaling via NFκB [28]. Hence, SKI-expressing tumor cells may evade NK cell-killing via inhibition of pro-inflammatory factors like TNFα. In line with the contribution of SKI to *MICA* gene regulation, ChIPseq analyses of the AML cell line HL60 revealed an enrichment of SKI in the promoter region of *MICA,* which was diminished upon SKI knockdown, confirming specificity (Figure 3C). 

**Figure 4:**
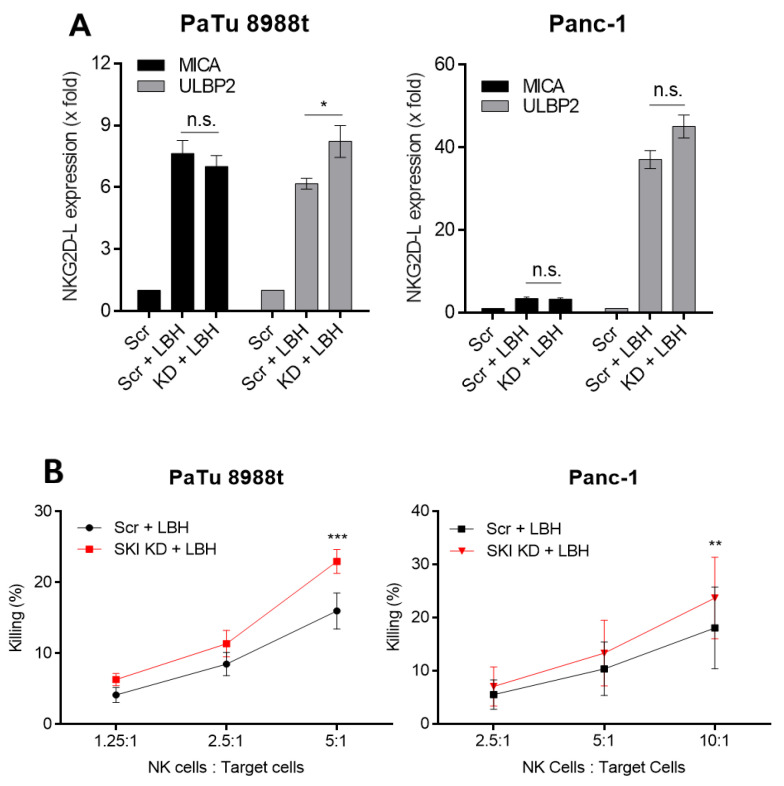
Effect of SKI knockdown and LBH treatment on NKG2D-L expression and NK cell-mediated killing. (**A**) siRNA-mediated SKI knockdown was performed for 24 h followed by 100 nM LBH589 and *MICA* and *ULBP2* were measured in PaTu8988t (**left panel**) and Panc-1 (**right panel**) by qRT-PCR. (**B**) 24 h after SKI knockdown and LBH treatment, NK cell-mediated killing of tumor cells was measured by flow cytometry. Data are the mean of three to five independent experiments ± SEM. Statistical significance was calculated using 1-way and 2-way ANOVA analyses (* *p* < 0.05, ** *p* < 0.01, *** *p* < 0.001, n.s.: not significant).

Next, both SKI wildtype and SKI knockdown cells were treated with LBH and the impact on the inducible expression of *MICA* and *ULBP2* was analyzed by qRT-PCR. Interestingly, we observed in knockdown cell lines a significantly higher induction of *ULBP2* transcription, which was in line with enhanced NK cell-dependent killing of PaTu8988t cells (Figure 4A,B). These data suggest that SKI interferes with the HDACi-dependent *ULBP2* upregulation on these pancreatic cancer cells. However, the HDACi-dependent induction of *MICA* remained unaltered in SKI wildtype cells and upon knockdown (Figure 4A). LBH-treated SKI-deficient Panc-1 cells were more efficiently killed than wildtype cells. However, no significant upregulation of *ULBP2* or *MICA* could be detected, suggesting that SKI suppresses NK cell immunosurveillance also by NKG2D/NKG2D-L-independent mechanisms.

In conclusion the combination of HDACi with NK cell-based immunotherapy is an attractive treatment option for pancreatic tumors. Therapy may be further improved by additional inhibition of SKI oncogenic activity, for example, via re-expression of NKG2D-L or pro-inflammatory factors leading to an improved recruitment and activation of NK cells to the TME. Further studies are needed to identify the crucial factors mediating the SKI repressive function in NK cell-killing.

Recent approaches to treat PDAC patients with NK cell-based immunotherapy showed promising results. One example is the adoptive transfer of ex vivo-expanded and antibody-activated NK cells resulting in IFNγ secretion and significant anti-tumor activity against pancreatic cancer cells [29]. Clinical trials have illustrated that the combination of allogeneic NK cell immunotherapy with percutaneous irreversible electroporation enhances the progression-free survival and overall survival in stage III PDAC and extends the overall survival in stage IV PDAC [30]. Recently, it was shown using a PDAC mouse model that the anti-tumor activity of gemcitabine chemotherapy was mediated through an increase of tumor-infiltrating NK cells and the decrease of myeloid-derived suppressor cells [31]. Interestingly, it was reported that gemcitabine, like HDACi, also modulates the expression of NKG2D-L on tumor cells further supporting NK cell-mediated anti-tumor effects.

HDACi may also impact directly on NK cells, for example, via up-regulation of NKG2D and stimulation of IFNg release and toxicity in NK-tumor cell co-cultures [32]. However, HDACi monotherapy was already tested in clinical trials but received rather disappointing results [33]. Our data suggest that the combination of HDACi with NK cell-based immunotherapy, such as adoptive NK cell transfer may be beneficial for PDAC patients. Strategies including the combination of epigenetic approaches or radiation with immune therapy are under investigation [34].Thus, HDACi may be used to change the immunosuppressive TME and demask the tumor cells for immunotherapeutic approaches for example, with activated transferred NK cells. However, a better understanding and strategies to overcome the immunosuppressive TME diminishing NK cell activity for example, via targeting the oncoprotein SKI, is mandatory for further studies. Taken together, there is evidence for the potential of NK cell-based therapies for PDAC. However the complex interactions of NK cells with the immunosuppressive TME of PDAC are only poorly understood and require further research.

## 3. Material and Methods

### 3.1. Isolation of Primary NK Cells and Culture of Tumor Cell Lines

Cell lines: The human pancreatic cancer cell lines Panc-1 and PaTu8988t cells were purchased from ATCC. The cells were cultured in DMEM with 10% FCS and 1% P/S at 37 °C and 5% CO_2_. 

NK cells: Leukocytes from leukoreduction system chambers of healthy donors were provided by the blood bank of the University Hospital of Giessen and Marburg. Peripheral blood mononuclear cells (PBMCs) were purified by ficoll density centrifugation followed by a centrifugation step at 10 g for 5 min to deplete the sample of monocytes. NK cells were then isolated using a negative selection NK cell isolation kit (Miltenyi Biotec, Bergisch Gladbach, Germany). Isolated NK cells were cultured in IMDM medium with 10% FCS, 1% P/S, 100 ng/mL IL-15 and 200 U/mL IL-2 and rested at 37 °C overnight before killing assays were performed. 

### 3.2. Killing Assay

For killing assays, target cells were stained with 5 µM CellTracker^TM^ Violet BMQC (Invitrogen, Carlsbad, CA, USA) fluorescent dye in serum-free medium at 37 °C for 45 min. Then, cells were washed twice in complete medium and seeded accordingly in IMDM medium with 10% FCS and 1% P/S in U-shaped 96-well plates. NK cells were added to the target cells in ratios ranging from 1.25:1 to 10:1. The cells were co-cultured for 3 h before they were harvested and centrifuged at 300 g for 5 min. The supernatant was discarded and the cells were resuspended in 200 µL PBS before they were stained with 100 ng 7-AAD (Biolegend, San Diego CA, USA). Cell death was measured by flow cytometry using a FACS Canto II cytometer (BD Bioscience, Heidelberg, Germany) and analyzed by FACS Diva software and FlowJo (version 10.6.1, (BD Bioscience, Heidelberg, Germany).

### 3.3. Detection of NKG2D-L on Tumor Cells

Tumor cells were treated with 25 nM LBH589 (Selleckchem, München, Germany), 2.5 mM VPA (Sigma-Aldrich, Darmstadt, Germany) or solvent DMSO (Sigma-Aldrich, Darmstadt, Germany) for 16 to 72 h before they were collected. The cells were centrifuged at 300 g for 5 min, the supernatant was discarded and the cells were resuspended in 200 µL PBS. Samples were then split in two and one half was incubated with 10 ng/100 µL sample anti-ULBP-2/5/6 PE-conjugated antibody (R&D Systems, Wiesbaden, Germany) and the other half with the corresponding IgG2a control (Biolegend, Koblenz, Germany) for 30 min on ice in the dark. The cells were washed with PBS and then analyzed by flow cytometry. Alternatively, cells were incubated with 0.15 µg/100 µL sample recombinant human NKG2D Fc chimera protein (R&D systems) or corresponding recombinant IgG1 Fc protein (R&D Systems, Wiesbaden, Germany) for 30 min on ice. Cells were washed with PBS and then incubated with AF647-coupled anti-human IgG Fc antibody for 25 min. Cells were washed with PBS and then analyzed by flow cytometry. Data were analyzed using FloJo software (BD Bioscience, Heidelberg, Germany). 

### 3.4. Quantitative Real-Time PCR (qRT-PCR) of NKG2D-L

Tumor cells were treated with 100 nM LBH589 for 16 to 72 h before cells were harvested and mRNA was isolated using the NucleoSpin RNA isolation kit according to the manufacturer’s instructions (Macherey-Nagel). Absolute SYBR Green Mix (ThermoFisher Scientific, Schwerte, Germany) was used with the following primers: ULBP2_for 5′-GCCGCTACCAAGATCCTTCT-3′, ULBP2_rev 5′-GCAAAGAGAGTGAGGGTCGG-3′, MICA_for 5′-CTGCAGGAACTACGGCGATA-3′, MICA_rev 5′-CCCTCTGAGGCCTCGCT-3′, L27_for 5′-AAAGCTGTCATCGTGAAGAAC-3′ and L27_rev 5′-GCTGTCACTTTGCGGGGGTAG-3′. The qPCR reactions were run in technical triplicates in a Thermo Cycler Mx3005P (Stratagen) using the following protocol: Initial step of 95 °C for 15 min, then 40 cycles of 95 °C for 15 s, 60 °C for 20 s and 72 °C for 15 s, followed by a denaturation step at 95 °C for 60 s and a melting curve analysis. The relative expression level of genes of interest was calculated by the ΔΔCt-method, with each target normalized to L27. Biological replicates were used to calculate standard deviations.

### 3.5. SKI Knockdown Using siRNA and ChIP-Seq

4 × 10^5^ Panc-1 or PaTu8988t cells were seeded in 6-well plates. The next day, cells were transfected with 100 pmol siRNA using Lipofectamine according to the manufacturer’s instructions (Thermo Fisher Scientific, Schwerte, Germany) and were harvested 24 h later. Human siGENOME SKI siRNA SMARTpool (M-003927-02-0005) and non-targeting siRNA #5 (D-001210-05) were purchased from Horizon Discovery (Waterbach, UK). ChIP-seq data were collected [28] and bioinformatical analysis are described there in the Materials & Methods section. 

### 3.6. Western Blot Analysis

For cell lysis, cells were pelleted and then lysed in RIPA buffer (50 mM Tris-Base, pH 7.4; 150 mM NaCl; 1 mM EDTA; 1% NP-40; 0.25% sodium deoxycholate and protease inhibitors (Roche, Rotkreutz, Germany) for 30 min on ice. The samples were centrifuged at 13,000 g at 4 °C for 15 min and the supernatant was collected for SDS-PAGE and immunoblotting. The following antibodies were used: anti-SKI (G8, sc-33693, Santa Cruz Biotechnology, CA, USA) in 10% Blocking Reagent (11096176001, Roche) and anti-β-Actin (a1978, Sigma-Aldrich, Taufkirchen, Germany). Detection was performed with horseradish peroxidase-conjugated secondary antibodies (DAKO, Hamburg, Germany) using Amersham ECL Plus (GE Healthcare, Freiburg, Germany).

### 3.7. Statistical Analysis 

Statistical significance was calculated using GraphPad Prism Software (GraphPad, La Jolla, CA, USA). 1-way and 2-way ANOVA, Sidak’s multiple comparisons test and Student’s *t*-test were applied as indicated in the figure legends.

## 4. Conclusions

In conclusion, the combination of HDACi with NK cell-based immunotherapy is an attractive treatment option for pancreatic tumors, specifically for patients with high SKI protein levels to overcome SKI-mediated immune evasion. 

## Supplementary Materials

The following are available online at https://www.mdpi.com/2072-6694/12/10/2857/s1, Figure S1: NKG2D-L (A) and ULBP2/5/6 (B) expression on tumor cells after treatment with LBH589 and VPA; Figure S2: Western blots of SKI/ß-ACTIN detection in PaTu899t lysate and Panc-1 lysate.
