# Supplementary material for: The Oncoprotein SKI Acts as A Suppressor of NK Cell-Mediated Immunosurveillance in PDAC
Source: Cancers (Basel). 2020 Oct 3;12(10):2857. doi: 10.3390/cancers12102857 (PMC7601115; doi:10.3390/cancers12102857)
Supplement: Supplementary file 1 [file cancers-12-02857-s001.pdf]

cancers

MDPI

Article

The Oncoprotein SKI Acts as A Suppressor of NK Cell-Mediated Immunosurveillance in PDAC

Viviane Ponath, Miriam Frech, Mathis Bittermann, Reem Al Khayer, Andreas Neubauer, Cornelia Brendel and Elke Pogge von Strandmann

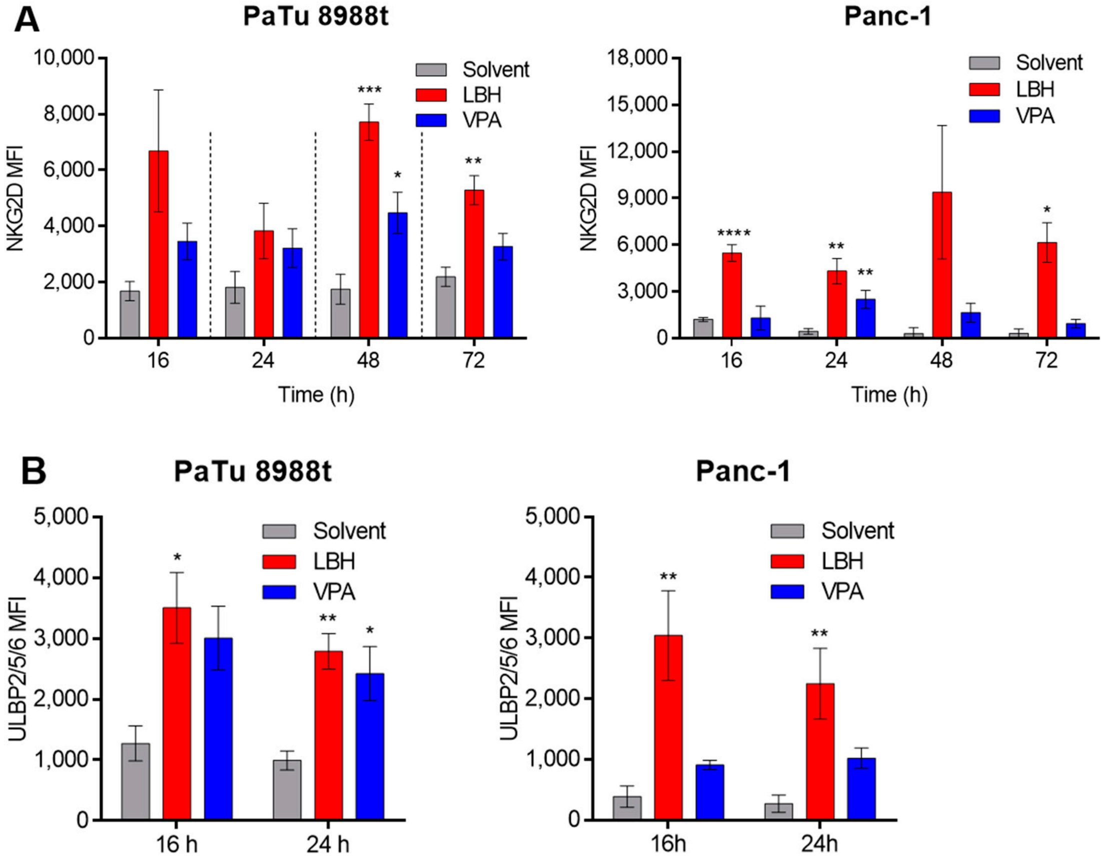

Figure S1: NKG2D-L (A) and ULBP2/5/6 (B) expression on tumor cells after Table 589. and VPA. PaTu8988t and Panc-1 cells were treated with 25 nM LBH589 or 2.5 mM VPA for 16 or 24 h. Then, protein surface expression was measured by flow cytometry. Data are the mean of four to eight independent experiments ± SEM. Statistical significance was calculated using 1-way ANOVA (* $p < 0.05$, ** $p < 0.01$, *** $p < 0.001$, **** $p < 0.0001$).

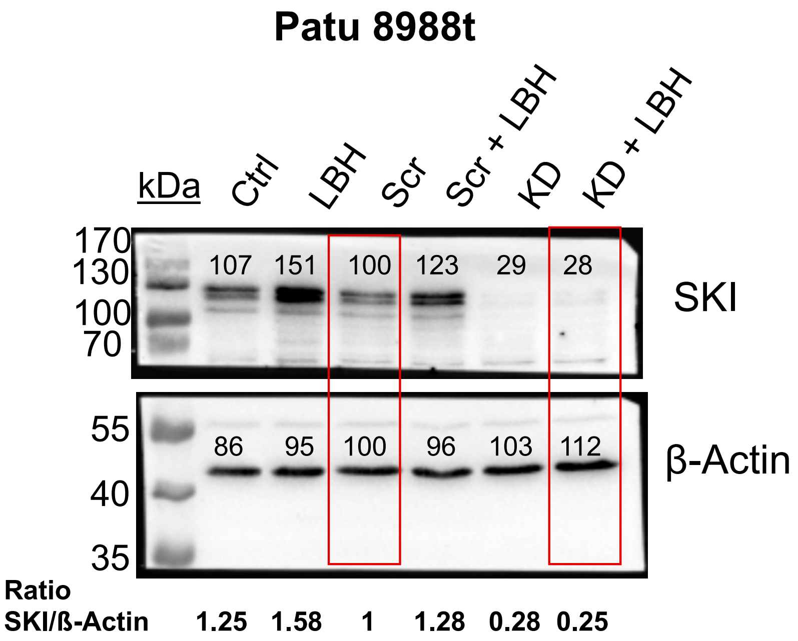

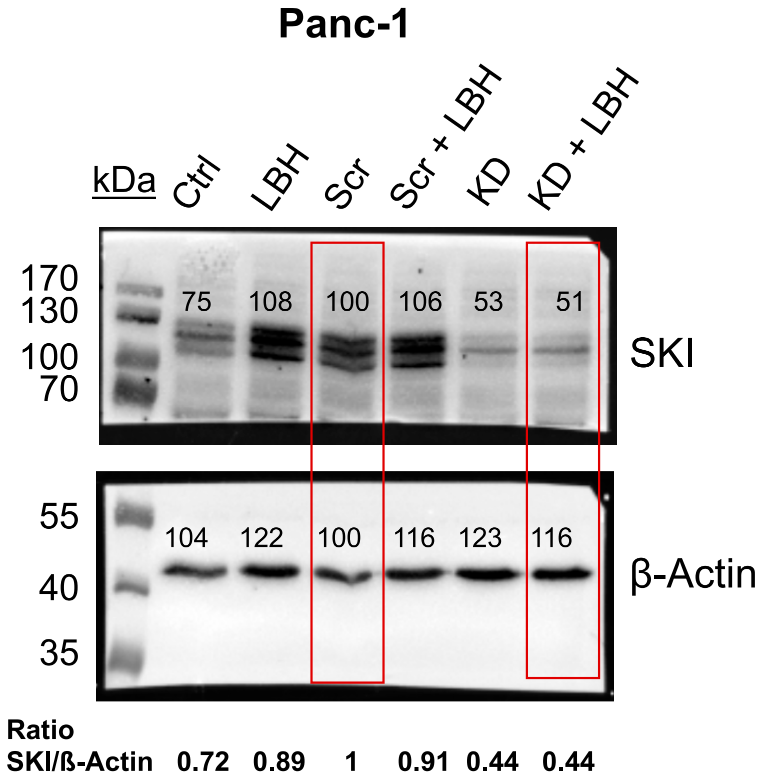

Figure S2: Western blots of SKI/ß-ACTIN detection in PaTu899t lysate and Panc-1 lysate (see Figure 2A for cut-out). Quantification was performed using image Lab(TM) Version 6.0.1, (Bio-Rad Laboratories, Inc., Feldkirchen, Germany.)
